# Bisphenols in daily clothes from conventional and recycled material: evaluation of dermal exposure to potentially toxic substances

**DOI:** 10.1007/s11356-024-34904-4

**Published:** 2024-09-06

**Authors:** Martina Jurikova, Darina Dvorakova, Kamila Bechynska, Jana Pulkrabova

**Affiliations:** Faculty of Food and Biochemical Technology, Department of Food Analysis and Nutrition, University of Chemistry and Technology (UCT), Prague, Technicka 5, 166 28, Prague, Czechia

**Keywords:** Bisphenol A, BPA structural analogues, Textile, Dermal exposure, UHPLC-MS/MS, Endocrine disruptors

## Abstract

Given the increasing concern about chemical exposure from textiles, our study examines the risks of dermal exposure to bisphenol A (BPA), bisphenol S (BPS), bisphenol B (BPB) and bisphenol F (BPF) from conventional and recycled textiles for adults, aiming to obtain new data, assess exposure, and evaluate the impact of washing on bisphenol levels. A total of 57 textile samples (33 from recycled and 24 from conventional material) were subjected to ultrasound-assisted extraction (UAE) followed by ultra-high performance liquid chromatography with tandem mass spectrometry analysis (UHPLC-MS/MS). The BPA and BPS concentrations varied widely (BPA: < 0.050 to 625 ng/g, BPS: 0.277–2,474 ng/g). The median BPA content in recycled textiles (13.5 ng/g) was almost twice as high as that of 7.66 ng/g in conventional textiles. BPS showed a median of 1.85 ng/g in recycled textiles and 3.42 ng/g in conventional textiles, indicating a shift from BPA to BPS in manufacturing practices. Simulated laundry experiments showed an overall reduction in bisphenols concentrations after washing. The study also assessed potential health implications via dermal exposure to dry and sweat-wet textiles compared to a tolerable daily intake (TDI) of 0.2 ng/kg bw/day for BPA set by the European Food Safety Authority (EFSA). Exposure from dry textiles remained below this threshold, while exposure from wet textiles often exceeded it, indicating an increased risk under conditions that simulate sweating or humidity. By finding the widespread presence of bisphenols in textiles, our study emphasises the importance of being aware of the potential risks associated with recycling materials as well as the benefits.

**Supplementary Information:**

The online version contains supplementary material available at 10.1007/s11356-024-34904-4.

## Introduction

At a time when environmental health is attracting increasing public and scientific attention, bisphenol A (BPA) stands out as one of the main chemicals of concern. As the primary representative of the bisphenols group and a proven endocrine disruptor, BPA is a ubiquitous environmental pollutant due to its extensive use in polycarbonate plastics, epoxy resins and various consumer products. Its detection across all significant environmental matrices – from aquatic systems to soil, air and dust – emphasises the widespread industrial application of the compound and the resulting ease with which it penetrates both ecosystems and the human body (Abraham and Chakraborty 2020; Ma et al. 2019).

Given its extensive production and widespread distribution in the environment, BPA has been extensively researched, with many toxicological studies demonstrating various adverse effects on human health. Most of these effects are due to their influence on the endocrine system, particularly estrogen, androgen and thyroid hormone receptors, leading to disturbances in various body systems (Ma et al. 2019). Studies also report adverse effects on the reproductive system (Kawa et al. 2021; Vitku et al. 2015), metabolic disorders are leading to obesity and associated diseases such as type 2 diabetes (Shankar and Teppala 2011), cardiovascular (Shankar and Teppala 2012) and liver diseases (Lee et al. 2014; Verstraete et al. 2018), immune disorders contributing to autoimmune diseases (Chailurkit et al. 2016) and adverse effects on child development, including prenatal onset of puberty, mental development and neurodevelopmental diseases (Berger et al. 2018; Chen et al. 2018; Jensen et al. 2019; Perera et al. 2012, 2016). In addition, various studies have linked BPA to different types of cancer, emphasising its potential health hazards (Dumitrascu et al. 2020; Leung et al. 2017; Tse et al. 2017; Zhang et al. 2014). While structural analogues are gradually being used as substitutes for BPA, detailed studies on their effects are less common. However, new research suggests that these substitutes may have similar adverse health effects as BPA (Catenza et al. 2021; den Braver-Sewradj et al. 2020; Qiu et al. 2018; Rochester and Bolden 2015; Zhang et al. 2018). Despite the extensive list of potentially harmful effects of bisphenols, the consequences on human health are still not fully understood (Ma et al. 2019).

As a result of the growing awareness of the dangers of BPA, its use is regulated, unlike its structural analogues, and is already restricted by law for some purposes. In the European Union, BPA is banned in the manufacture of cosmetics (Regulation (EC) No 1223/2009 of the European Parliament and of the Council) and polycarbonate baby bottles (Commission Implementing Regulation (EU) No 321/2011). As a particularly vulnerable group, infants are protected by the directive on toy chemicals, which sets the migration limit for BPA at 0.04 mg/L (Commission Directive (EU) 2017/898 2017). Another important document related to BPA is a new scientific opinion issued by the European Food Safety Authority (EFSA) in 2023, which concludes that the tolerable daily intake (TDI) of 4 μg/kg bw/day from 2015 should be lowered to a new level of 0.2 ng/kg bw/day, as BPA has been shown to have harmful effects on the immune system and affects cells involved in the development of autoimmune diseases. Their alarming conclusion is that this new TDI level would be exceeded by 2–3 times in all age groups, based on observed dietary exposure levels from 2015 (EFSA Panel on Food Contact Materials, Enzymes and Processing Aids 2023). In contrast, the United States Environmental Protection Agency (U. S. EPA) has established a Reference Dose (RfD) for BPA at 50 μg/kg bw/day, a level set in 1988 based on studies that primarily focused on reproductive and developmental effects, which remains significantly higher than the newly proposed TDI by EFSA (U. S. EPA 1988). However, similar reference doses or tolerable daily intake levels for other bisphenols such as BPS, BPB, and BPF have not been comprehensively established by regulatory authorities like the US EPA or EFSA, as the toxicological data on these substances are still limited, and their health effects remain under-researched compared to BPA.

Specifically for humans, the dietary route of BPA exposure is the most significant (more than 90% of BPA exposure); the dermal route, together with inhalation represent minor sources (EFSA Panel on Food Contact Materials, Enzymes and Processing Aids 2015; Geens et al. 2012). However, it appears that BPA absorbed dermally is eliminated from the body differently than BPA ingested through food, bypassing first-pass metabolism (Martínez et al. 2018). After dietary intake, BPA is absorbed from the gastrointestinal tract and biotransformed mainly into glucuronide conjugates to reduce its biological activity and then excreted from the organism with a half-life of less than 6 h (Dekant and Völkel 2008; Ma et al. 2019). However, Liu and Martin (2017) have shown that BPA absorbed through the skin is excreted from the body over a more extended period (detectable BPA concentrations in urine for more than a week) and that more unconjugated BPA also circulates in the serum during this process compared to oral exposure. This characteristic emphasises the importance of investigating the dermal route of exposure to BPA rather than overlooking it.

An important source of dermal exposure to BPA is often attributed to handling receipts. However, a growing number of studies observe this substance's presence in clothing, where the exposure time is significantly higher. In the textile industry, it is used as an intermediate for the production of dyes and antioxidants to increase the stability of fibres (European Commission Scientific Committee on Consumer Safety 2020; Herrero et al. 2023; Wang et al. 2019). The presence of BPA is often associated with textiles made from synthetic fibres, but it has also been found in 100% cotton textiles (Freire et al. 2019; Herrero et al. 2023; Li and Kannan 2018; Wang et al. 2019; Xue et al. 2017). These initial studies have confirmed the presence of BPA in textiles and its structural analogues, mainly BPS, to which manufacturers are switching, which are likely to have similar toxicological effects on human health. This hypothesis is the subject of a growing number of scientific publications that progressively confirm this claim (den Braver-Sewradj et al. 2020; İyİgÜndoĞdu et al. 2020; Mokra et al. 2017; Qiu et al. 2018; Rochester and Bolden 2015; Zhang et al. 2018). Given the recent reduction of the TDI for BPA, our research question focuses on whether the presence of bisphenols in textiles, through prolonged daily contact, could significantly contribute to exceeding this newly established, low TDI level.

Despite the increasing volume of research on bisphenols and their ubiquity in various environmental matrices, there is still a notable gap in the literature on their occurrence in textiles. To date, only five studies have directly addressed this topic, with four of them including the BPA structural analogues (Freire et al. 2019; Herrero et al. 2023; Li and Kannan 2018; Wang et al. 2019; Xue et al. 2017). In addition, there is no published study on bisphenols in recycled fabric textiles, which is becoming increasingly popular due to growing environmental awareness and sustainability efforts.

The present study aims to (a) extend the limited knowledge on the presence of bisphenols represented by bisphenol B (BPB), bisphenol F (BPF) and bisphenol S (BPS) in 31 T-shirts and 23 pairs of socks, focusing on the differences between conventional (24 items) and recycled textiles (30 items), (b) based on the obtained data conduct a dermal exposure assessment for dry and sweat-moistened textiles, reflecting real-life scenarios, and (c) perform a preliminary evaluation of the impact of washing on bisphenols levels in textiles.

## Materials and methods

### Chemicals and other materials

The following analytical standards were used in our study: BPA (99% purity), BPB (98% purity), BPF (98% purity) and BPS (99.7% purity), all supplied by Sigma Aldrich® (St. Louis, MO, USA). As internal standards, isotopically labelled standards (internal standards, IS) d6-BPA (98% purity) and d8-BPS (98.4% purity) were employed, both supplied by Chiron (Norway). Catalogue numbers of utilised standards are presented in Text S1. Mixtures of the individual standards in the desired composition and concentration were prepared in methanol (MeOH, high-performance liquid chromatography (HPLC) grade, Honeywell, Riedel-de Haën™, Germany).

Ethyl acetate (EtOAc) and acetonitrile (MeCN) were used, both HPLC grade supplied by Honeywell (Riedel-de Haën™, Germany), together with dichloromethane (DCM; HPLC grade, SupraSolv®, Supelco®, (USA)). In addition, acetic acid (CH_3_COOH; 99%, Penta, Czech Republic) was used to prepare the mobile phases.

Synthetic sweat (acidic solution) was prepared according to the International Standard Organisation (ISO105-E04-2008E) using grade 3 water (Milli-Q® Integral 3 System, Millipore, France) according to ISO 3696 and contained L-histidine monohydrochloride monohydrate (Sigma Aldrich, USA), sodium chloride (Lach-Ner, Czech Republic), sodium dihydrogen phosphate dihydrate (Penta, Czech Republic), for exact composition see Supplementary material (Table S1). NaOH (Penta, Czech Republic) solution was used to adjust the pH of the synthetic sweat; details are given in Table S1 in the Supplementary material.

Other materials used are solid-phase extraction (SPE) columns (HLB 6 cc (150 mg), Oasis®, Ireland) and microfilters Spin-X, 0.22 μm nylon (Costar®, USA). A gentle detergent gel for sensitive baby skin was used in an experiment simulating the washing process.

### Samples collection and preparation

The clothing samples were collected in two rounds in 2022. In the first round (March/April), we focussed on conventional textiles, gathering 24 items (14 T-shirts, 10 socks). In the second round (October/November), we collected 30 items (17 T-shirts, 13 socks) made from recycled materials, with each sample containing at least 22% recycled fibre material. The items were purchased from international clothing (mainly fast fashion) or sports fashion chains. In addition to the target fabric type (conventional vs. recycled), the criterion was a price of up to 15 EUR per piece to cover clothes that are likely to be widely purchased. Detailed information on the samples' composition, colour and origin can be found in Supplementary material (Table S2).

New, unwashed textile samples were cut into small pieces with scissors, washed in acetone and stored in a polypropylene (PP) tube in the dark for further processing.

### Sample extraction procedure

The extraction principle consists of the repeated extraction of textile samples in MeCN. Into a PP tube containing 1 g of homogenised sample, 20 mL of MeCN was added, and the sample was subjected to ultrasound-assisted extraction (UAE) for 2 h. The extract was then transferred to an evaporation flask, and an additional 20 mL of MeCN was added to the same sample, which was extracted for a further 2 h. The extracts obtained were combined in an evaporation flask (yielding 40 mL of extract) and then evaporated to dryness. The dry residue was then reconstituted in 450 µL MeCN and fortified with the IS mixture to a final volume of 500 µL. The mixture was then microfiltered and transferred to vials for further analysis.

Throughout the process of method optimisation, key parameters were refined: (a) selection of extraction solvent and (b) determination of extraction time and number of repetitions. In the first experiment (a), a sample of a recycled fabric bag was extracted in the test solvent (MeOH, MeCN, DCM and EtOAc) for 2 h using ultrasound, each solvent being in three repetitions. In the second experiment (b), the textile used for the test was extracted in triplicate: 20 mL of MeCN was added to 1 g of the sample and then extracted for 2 h. The extract obtained was transferred to an evaporation flask, evaporated, and further processed as described above in the complete extraction procedure. A further 20 mL of solvent was added to the sample, and a second extraction was performed. The extract was processed similarly to the previous step. This procedure was repeated so that three consecutive extractions were performed for each sample, each being analysed separately.

### Laundry simulation experiment

The design of the laundry experiment was inspired by Wang et al. (2019) and conducted with modifications. A 12 × 12 cm area was cut out as one piece from selected garments and placed in a 500 mL Erlenmeyer flask with 400 mL of deionised water at 30 ºC and 0.4 g of gentle detergent for sensitive baby skin. The flasks were then placed onto an orbital shaker and agitated (200 rotations per minute) for one hour to simulate the washing process. Subsequently, the wash water was discarded, and the flasks were refilled with 400 mL of fresh water to simulate the rinsing step of a conventional laundry cycle. The samples were returned to the orbital shaker and shaken for 30 min. Afterwards, the rinsing water was discarded, and the fabric was carefully wrung out using a glass rod to prevent external contamination. The samples were then air-dried at room temperature in a controlled environment to avoid further contamination. After drying, the fabric squares were processed and stored according to the protocols described in chapter "Samples collection and preparation".

### Sweat leaching experiment

To test the potential transfer of bisphenols from the fabric to the synthetic sweat, four samples with previously identified bisphenols content were selected, cut to a size of 4 × 2 cm and weighed. The SPE columns were preconditioned with 4 mL of a mixture of MeOH:DCM (1:1, v/v), followed by 4 mL of MeOH and 4 mL of deionised water. Subsequently, the fabric samples were rolled into the columns to avoid overlapping and to ensure proper contact with the synthetic sweat. Then, 6 mL of synthetic sweat was added, and the fabrics were soaked for 2 h. The contents of the columns were then emptied under pressure, and the fabric was removed. The column was then washed with 8 mL of deionised water. The columns were then placed in centrifugation tubes and centrifuged at 10,000 RPM for 10 min. The analytes were eluted from the column with 5 mL of MeOH:DCM (1:1, v/v) into an evaporation flask. Then 25 µL of the IS mixture (1,000 ng/mL) was added to the eluate, the solvent evaporated, and the dry residue reconstituted with 500 µL of MeOH.

The method was validated by analysing synthetic sweat with a known addition of bisphenols in six replicates. The characteristics of the method obtained are listed in Table S3 in Supplementary material.

### Instrumental analysis

The target analytes were identified and quantified using an ultra-high performance liquid chromatograph (UHPLC) 1290 Infinity II LC (Agilent Technologies, USA) coupled to a Sciex QTRAP 6500 + mass spectrometer (MS), a hybrid triple quadrupole/linear ion trap type, with electrospray operating in negative mode (ESI).

The injected sample volume was 5 µL. A Kinetex Biphenyl column (100 × 2 mm; 1.7 µm) (Phenomenex, USA) heated at 40 ºC was used to separate the analytes. The mobile phases consisted of 0.05% CH_3_COOH in deionised water (A) and 0.05% CH_3_COOH in MeOH (B), with a gradient described in detail in Supplementary material (Table S4). Other UHPLC-MS/MS parameters are described in Table S5 (in Supplementary material). The UHPLC-MS/MS chromatogram of the solvent standard is presented in Fig. S1 (Supplementary material), and the chromatogram of a sample made of 100% recycled polyester (T20) is shown in Fig. S2 (Supplementary material). The analysis time per sample was 8 min.

### QA/QC

The method's performance characteristic in terms of repeatability was evaluated by analysing a sample of recycled fabric bags in six replicates. Instead of the recovery rate, the method's efficiency was assessed by selecting the time required for sufficient extraction and the number of repetitions, as described in chapter "Sample extraction procedure".

Calibration was based on a mixture of four bisphenols (BPA, BPB, BPS, BPF) at concentrations of 0.1, 0.5, 1, 5, 10 and 50 ng/mL, achieving a coefficient of determination (R2) of at least 0.99 for all calibration points. Samples with concentrations outside the calibration range were re-analysed after appropriate dilution. Isotopically labelled standards d6-BPA and d8-BPS were used as IS to facilitate the identification and quantification of the analytes. The limits of quantification (LOQs) for each compound were set based on the lowest calibration point of the analyte standard that achieved a signal-to-noise ratio (S/N) greater than 10 for quantification and greater than 3 for the confirmatory transitions. Consequently, the LOQs were set at 0.05 ng/g for BPA and BPS and 0.5 ng/g for BPF and BPB. The limits of detection (LODs) were determined as 1/3 of the LOQs.

The laboratory equipment and chemicals, including SPE columns and wash gel, were confirmed free of the target analytes. However, a process blank performed with each set of extracted samples showed BPA ultra-trace levels between < 0.05 and 0.1 ng/g, subtracted from the sample results to correct any background interference. To assess the quality of the sampling and extraction process, every 10th sample was analysed in duplicate.

### Statistical analysis

Descriptive statistics were performed in Microsoft Excel 2019; the values below the LOQ were replaced by half of the LOQ value for these calculations. Log-transformed BPA and BPS concentrations were used to create boxplots for data visualisation. Statistical differences between the bisphenols concentrations in conventional and recycled fabric textiles and the samples before and after washing were assessed using t-tests in custom-built R-scripts. Correlations (Spearman’s rank correlation coefficient) between the cotton content and detected bisphenol concentrations were studied in MetaboAnalyst 5.0 (metaboanalyst.ca) using Pattern Search. Log-transformed data were used to ensure a normal distribution of the data. Statistical significance was set at a p-value < 0.05.

### Dermal exposure assessment calculations

The dermal exposure to bisphenols from textiles can be estimated by considering the exposure to dry and sweat-wet material. For dry material, we applied Eq. 1 as described by Xue et al. (2017):

1 $${EXP}_{derm} = \frac{C \times D \times SA \times {F}_{mig} \times {F}_{contact} \times {F}_{pen} \times T \times N}{BW}$$

where *EXPderm* stands for the daily dermal exposure dose (pg/kg of bw/day), *C* is the concentration of the analyte detected in the tested fabric (ng/g), *D* is the density of the textile (mg/cm^2^), *F*_*mig*_ is the migration rate of the chemicals on the skin per day (0.005, 1/day), *F*_*contact*_ is the fraction of contact area for the skin (without unit; 1), *F*_*pen*_ is the rate of penetration of chemicals into the body (without unit; 0.01), *T* is the time of contact between the textile and the skin (1 day), *N* is the mean number of events per day (assumed 1/day). In addition, two *SA* values (skin surface area in contact with the textile) were used – for the T-shirt and the socks. The value for the T-shirt was 7,600 cm^2^, calculated based on a men's size M according to the standard size charts, considering contact with the front and back of the T-shirt. The value for the socks was calculated based on the average size 39 (EU) and was 450 cm^2^. The exposure estimate was made for an adult weighing 70 kg, corresponding to the *BW* value (body weight).

The estimation of the dermal exposure dose to bisphenols through contact with sweat-soaked textiles (*EXPderm*_*wet*_, ng/kg bw/day) was performed using Eq. 2, as used by Wang et al. (2019):

2 $${EXPderm}_{wet}=\frac{MR\times SA\times {F}_{pen}}{BW}$$

where *EXPderm*_*wet*_ represents the daily dermal exposure dose (ng/kg bw/day), *MR* is the simulated migration fraction from the textile to the skin, *SA* is the skin surface in contact with the textile (7,600 cm^2^ for T-shirts and 450 cm^2^ for socks), *F*_*pen*_ is the penetration rate of chemicals into the body, and *BW* is the body weight (70 kg for an adult).

The *MR* value was calculated using data from the sweat leaching experiment, in which the detected bisphenol concentrations (ng) were divided by the area of the tested textiles (8 cm^2^). As there is disagreement on the actual value of *F*_*pen*_, two reported values were considered – 10% (0.1) and 46% (0.46), resulting in *EXPderm*_*wet-low*_ and *EXPderm*_*wet-high*_, respectively (Wang et al. 2019).

## Results and discussion

### Extraction procedure characteristics

The development of the extraction method was based on similar studies on this topic using UAE as the core procedure (Freire et al. 2019; Herrero et al. 2023; Li and Kannan 2018; Wang et al. 2019; Xue et al. 2017). MeCN was selected as the most suitable of the four solvents tested because it provided the highest amounts of analytes (BPA and BPS) detected in the test material, as documented in Fig. S3 (Supplementary material). We also determined the time and number of extraction repetitions to make the process efficient. As shown in Fig. S4 (in Supplementary material), the relative recovery after two extraction cycles of 2 h each was 95% for BPA and 97% for BPS, making this process the most suitable.

The monitored parameter of the method performance was repeatability, expressed as relative standard deviation (RSD, %), which was evaluated using a sample from a recycled fabric bag analysed in six replicates. As BPA and BPS were the only analytes detected in the material used for the validation, the RSDs were determined for them only: 16% for BPA and 20% for BPS, indicating suitable method reproducibility. Since the octanol–water partition coefficients (logKow) for the target bisphenols are similar (logKow BPS = 1.65; BPF = 2.91; BPA = 3.43; BPB = 4.13 (Catenza et al. 2021)), we assume satisfactory extraction efficiencies also for BPF and BPB, which were not detected in the test textile. The LOQs were set at 0.05 ng/g for BPA and BPS and 0.5 ng/g for BPF and BPB.

### Occurrence of bisphenols in textile

The results of the target bisphenols analysis (Table 1) show a widespread occurrence of BPA and BPS, although the concentrations vary considerably. These compounds were consistently detected in both T-shirt and sock samples, regardless of whether they were made from conventional or recycled fabrics. In contrast, BPF was only detected in one sample (sample T20; 100% recycled polyester; blue colour; country of origin: Czech Republic). BPB was not detected in any sample of the entire sample set; therefore, it was excluded from the comparison in Table 1 and further discussion.

**Table 1:** Summarised results of the analysis of BPA, BPS and BPF in samples of conventional and recycled fabric textiles

	Detection Rate [%]	Range [ng/g]	Median^a^ [ng/g]	Mean^a^ [ng/g]	SD [ng/g]
All samples (*n* = 57^b^)					
BPA	96	< 0.050–625	7.83	59.4	143
BPS	100	0.277–2,474	2.20	65.3	337
BPF	2	< 0.500–57.3	x	x	x
All conventional fabric samples (*n* = 24)					
BPA	92	< 0.050–54.0	7.66	12.3	13.5
BPS	100	< 0.050–2,474	3.42	149	507
BPF	0	x	x	x	x
All recycled fabric samples (*n* = 33)					
BPA	100	0.546–625	13.5	93.6	180
BPS	100	0.277–42.2	1.85	4.83	7.77
BPF	3	< 0.500–57.3	x	x	x
All T-shirt samples (*n* = 33^c^)					
BPA	94	< 0.050–603	5.85	58.9	148
BPS	100	0.277–78.9	1.61	6.21	14.6
BPF	3	< 0.500–57.3	x	x	x
All conventional fabric T-shirt samples (*n* = 14)					
BPA	84	< 0.050–54.0	6.12	13.4	17.1
BPS	100	0.562–78.9	1.41	9.62	21.3
BPF	0	x	x	x	x
All recycled fabric T-shirt samples (*n* = 19^d^)					
BPA	100	0.546–604	3.64	92.4	187
BPS	100	0.277–17.4	1.68	3.70	4.72
BPF	5	< 0.500–57.3	x	x	x
All socks samples (*n* = 24^e^)					
BPA	100	1.55–625	12.1	60.0	136
BPS	100	0.876–2,473	4.19	147	508
BPF	0	x	x	x	x
All conventional fabric socks samples (*n* = 10)					
BPA	100	5.39–21.1	8.87	10.7	4.97
BPS	100	3.40–2,474	10.5	343	743
BPF	0	x	x	x	x
All recycled fabric socks samples (n = 14^f^)					
BPA	100	1.55–625	16.6	95.3	170
BPS	100	0.876–42.21	3.11	6.36	10.4
BPF	0	x	x	x	x

SD = standard deviation, x = not calculated

^a^Samples with no detectable analytes were included in the calculation as ½ LOQ value

^b^57 samples made of 54 clothing items

^c^33 samples made of 31 T-shirt items (4 samples were from 2 T-shirts)

^d^19 samples made of 17 T-shirt items (as above)

^e^24 samples made of 23 socks (2 samples from 1 sock)

^f^14 samples made of 13 socks (as above)

The highest BPA concentration (625 ng/g) was found in recycled fabric socks (sample S12a; 49% recycled polyester, 48% cotton, 2% elastane, 1% recycled polyamide; white colour; country of origin: Turkey). Interestingly, a separate analysis of the grey part (sample S12b) showed an approximately twice as high concentration of BPA (298 ng/g). However, BPS concentrations in both parts of the socks were similar (4.21 ng/g in S12a and 4.93 ng/g in S12b). The differences in BPA concentrations between the sock parts could be due to differences in material composition, colouring and chemical treatment, which may influence BPA absorption. From the entire sample set, BPA was not detectable in only two samples, T-shirts T7 and T8, which were made of 100% cotton. For BPS, the highest concentration (2,474 ng/g) was again found in a sock sample, but this time from non-recycled material (sample S2; 76% cotton, 22% polyamide, 2% elastane; purple unicorn print; country of origin: Vietnam). The BPA concentration in the same sample was not as pronounced (7.69 ng/g).

**Fig. 1:**
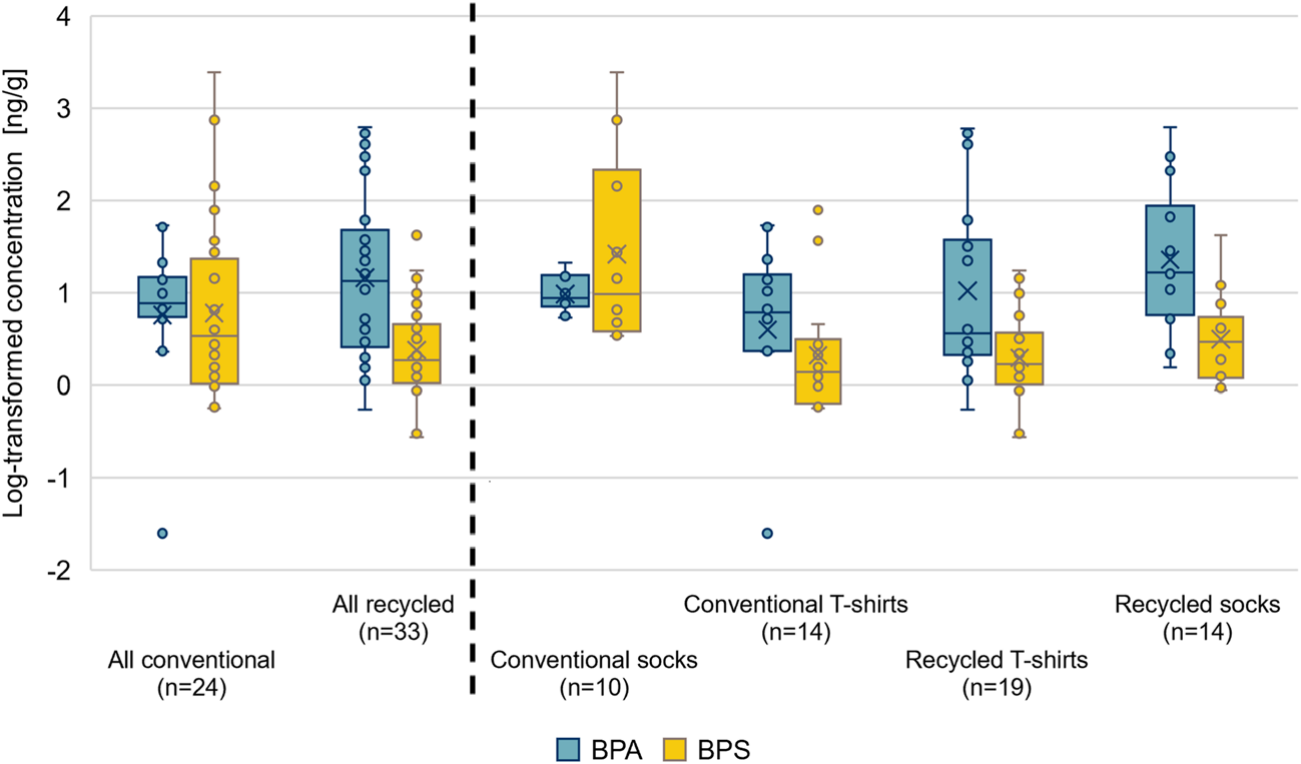
Log-transformed concentrations of BPA and BPS in *all conventional* and *recycled* fabric samples, together with detailed insight into each category – *conventional fabrics socks*, *conventional fabrics T-shirts*, *recycled T-shirts*, *recycled socks*. In the plot, the whiskers represent the minimum and maximum, the boxes represent the 25th and 75th percentiles, the horizontal line within the box represents the median (50th percentile), and the cross indicates the mean value. Values < LOQ were included as LOQ/2

As mentioned above, the detected concentrations of BPA and BPS showed a high variability (Fig. 1), especially for BPS in clothing samples from conventional fabric textiles, where the mean value was almost 44 times higher than the median value. BPA demonstrated a noticeable presence in the samples from recycled fabrics, with higher mean (93.6 ng/g) and median (13.5 ng/g) concentrations compared to conventional textiles (mean 12.3 ng/g and median 7.66 ng/g). However, the statistical analyses did not confirm a significant difference in the presence of BPA between recycled and conventional textiles (*p*-value = 0.0732), emphasising the importance of further research to understand these observations fully. The data suggest a consistent presence of BPA in recycled materials, possibly indicating extensive use in the past, its persistence during the recycling process, or higher levels of BPA in the source materials designated for recycling. These trends raise concerns regarding the possible accumulation of these compounds in recycled materials.

Conversely, samples from conventional fabric had higher mean (149 ng/g) and median (3.42 ng/g) concentrations of BPS compared to recycled textile fabrics (mean 4.83 ng/g and median 1.85 ng/g) but also showed a considerable discrepancy compared to BPA concentrations in conventional textiles (mean 12.3 ng/g, median 7.66 ng/g), indicating a possible shift in manufacturing practises. Although statistical tests could not confirm this difference as significant (*p* = 0.0790), the transition from BPA to BPS is also supported by the findings of Li and Kannan (2018), who reported that the median BPS concentration in pantyhose was 100 times higher than that of BPA. These observed trends are consistent with the hypothesis that manufacturing practises have evolved in response to the regulatory environment, shifting from BPA to substitutes such as BPS. However, recycled materials continue to show traces of the historical use of BPA. This shift towards BPA alternatives can be observed in various industries, for example, in the production of thermal paper (European Chemicals Agency 2020) and is also evidenced by some studies observing an increase of BPA analogues in the environment (Catenza et al. 2021; Liu et al. 2021). These findings emphasise the need for further research on the impact of fabric recycling processes on the concentration of these chemicals and their potential impact on human health and the environment.

**Fig. 2:**
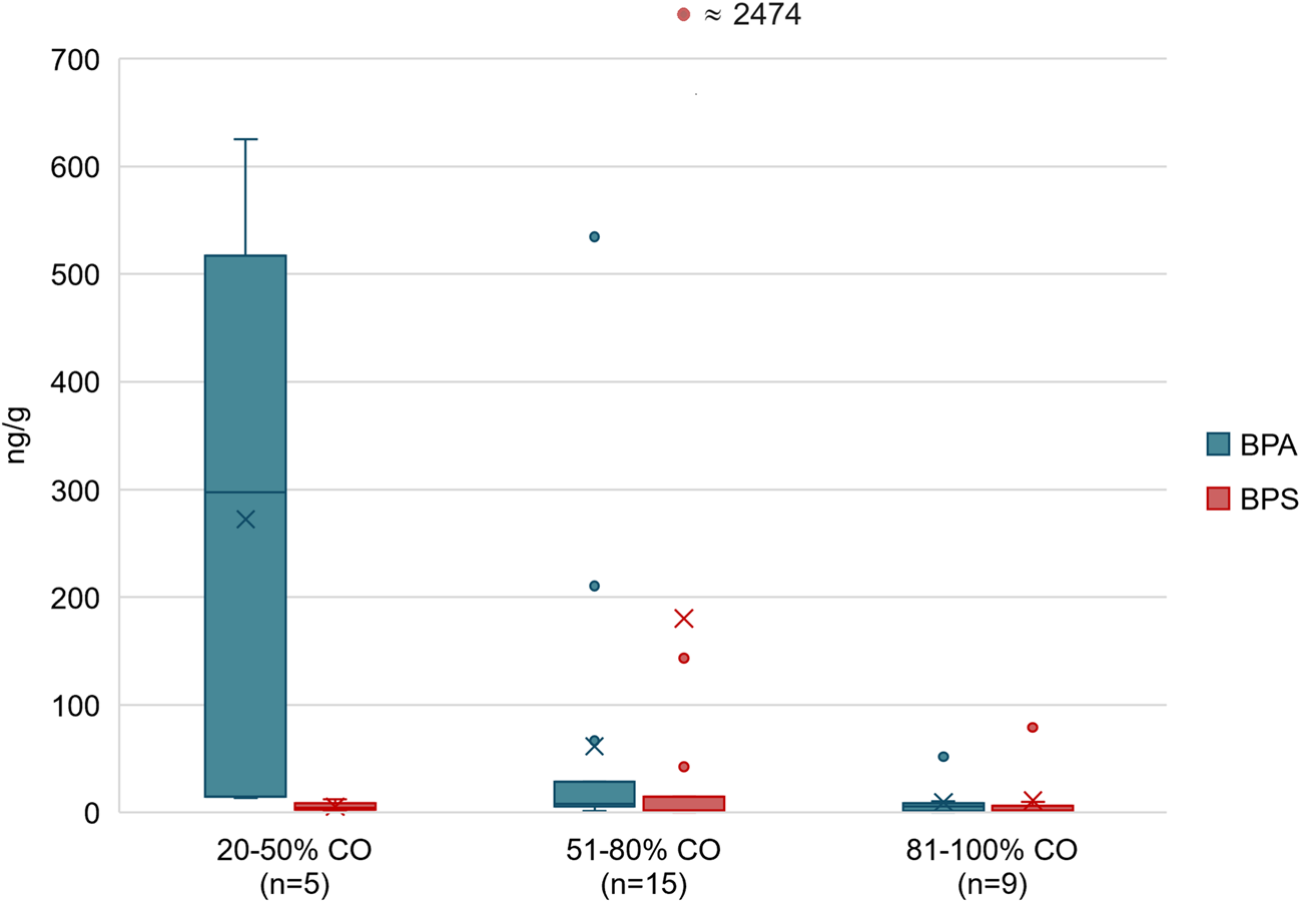
BPA and BPS concentrations (ng/g) in 29 textile samples according to the cotton content (CO) in %. The whiskers represent the minimum and maximum; the boxes represent the 25th and 75th percentiles; the horizontal line within the box is the median (50th percentile), and the cross indicates the mean. Values < LOQ were included as LOQ/2

In Fig. 2, we examined BPA and BPS concentrations in textile samples with varying cotton content, categorised as 20 to 50%, 51 to 80% and 81 to 100%. Our data suggest a pattern consistent with the results of other studies, indicating lower BPA concentrations in textiles with higher cotton content (Wang et al. 2019; Xue et al. 2017). The correlation between cotton content and BPA concentration was statistically confirmed (Spearman's rank correlation coefficient equal to -0.56, p-value < 0.01), while no trend was observed for BPS (Spearman's rank correlation coefficient -0.23, p-value = 0.21). This inconsistency underscores the complexity of bisphenol distribution within textiles, which may be influenced by factors beyond cotton content alone, such as manufacturing processes or other chemical treatments. Notably, Freire et al. (2019) reported higher median BPA levels in textiles made of 85% and > 90% cotton compared to those with lower cotton content, further emphasising the ambiguous nature of bisphenols in textiles. These observations support the need for a more comprehensive dataset and suggest that future research should investigate additional variables that may influence the absorption and distribution of bisphenols in textiles.

For context, Table 2 provides a comparative analysis of key parameters and results from previously published studies on the occurrence of bisphenols in textiles alongside our results. As BPB was not detected in our study, the comparison was limited to BPA, BPF and BPS. This comparison reveals that BPA and BPS were frequently detected in textiles, unlike other bisphenols. The detected concentrations varied considerably in the different studies, in some cases by several orders of magnitude, which is particularly evident in the study by Li and Kannan (2018). It is important to note that although BPB was not detected in our study, it was identified in the studies by Herrero et al. (2023) and Li and Kannan (2018), with a detection rate of 60% in the latter study. These results indicate diversity in the use and detection of bisphenols in textiles, which calls for further research to understand their distribution better.

**Table 2:** Comparison of the parameters (country, number of samples, sample type, method limit of detection (LOD) and LOQ in ng/g) and the results (detection rate in %, range and median in ng/g) of BPA, BPS and BPF in our study with published scientific studies

		Xue et al. (2017) Infant clothing (*n* = 77)	Li et Kannan (2018) Pantyhose (*n* = 36)	Freire et al. (2019) Socks (*n* = 32)	Wang et al. (2019) New, used clothing (*n* = 93)	Herrero et al. (2023) Pregnant woman, infant clothing (*n* = 120)	Our study (2024) T-shirts and socks (*n* = 54)
Country of purchase		USA	China, Japan, Korea, Chile, Portugal, USA	Spain	China	Spain	Czech Republic
BPA	LOD/LOQ	2.21	1.3	0.7	3.33	0.59	0.05
	Detection rate (%)	82	96	91	99	100	96
	Range	< 2.21–13,300	< 1.3–504	< 0.70–3,736	< 3.30–1,823	0.69–5,872	< 0.05–625
	Median	10.7	14.3	20.5	26.9	7.43	7.83
BPS	LOD/LOQ	0.74	0.3	x	0.53	0.12	0.05
	Detection rate (%)	53	100	x	43	94	100
	Range	< 0.74–394	< 0.3–2,190,000	x	< 0.53–536	< 0.12–981	0.28–2,474
	Median	1.02	1430	x	7.38	1.04	2.2
BPF	LOD/LOQ	14.71	12.5	x	ND	0.69	0.5
	Detection rate (%)	5.2	49	x	ND	73	2
	Range	< 14.7–194	< 12.5–1,280,000	x	ND	< 0.69–11,333	< 0.05–57.3
	Median	0.32	8.8	x	ND	1.01	x
BPB	LOD/LOQ	x	1.3	x	x	0.04	0.5
	Detection rate (%)	x	60	x	x	13	ND
	Range	x	< 1.3–7,230	x	x	< 0.04–44.1	ND
	Median	x	1.7	x	x	< 0.04	ND

*n* = number of samples, ND = not detected; x = not monitored

#### Laundry experiment

**Fig. 3:**
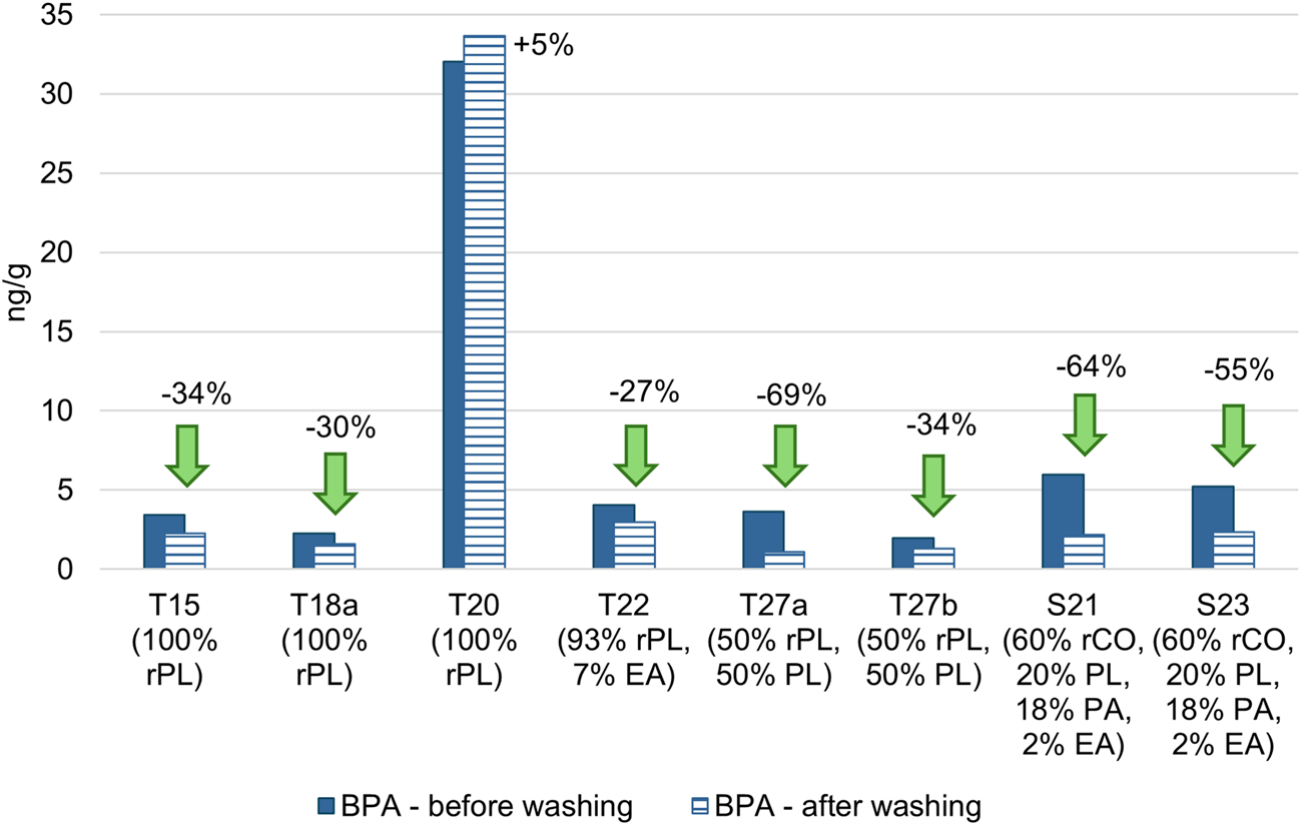
Decrease in BPA in new textiles after one wash cycle (a negative numerical value indicates a percentage decrease after a wash cycle, a positive value means an increase); rPL = recycled polyester, EA = elastane, PL = polyester, rCO = recycled cotton, PA = polyamide

**Fig. 4:**
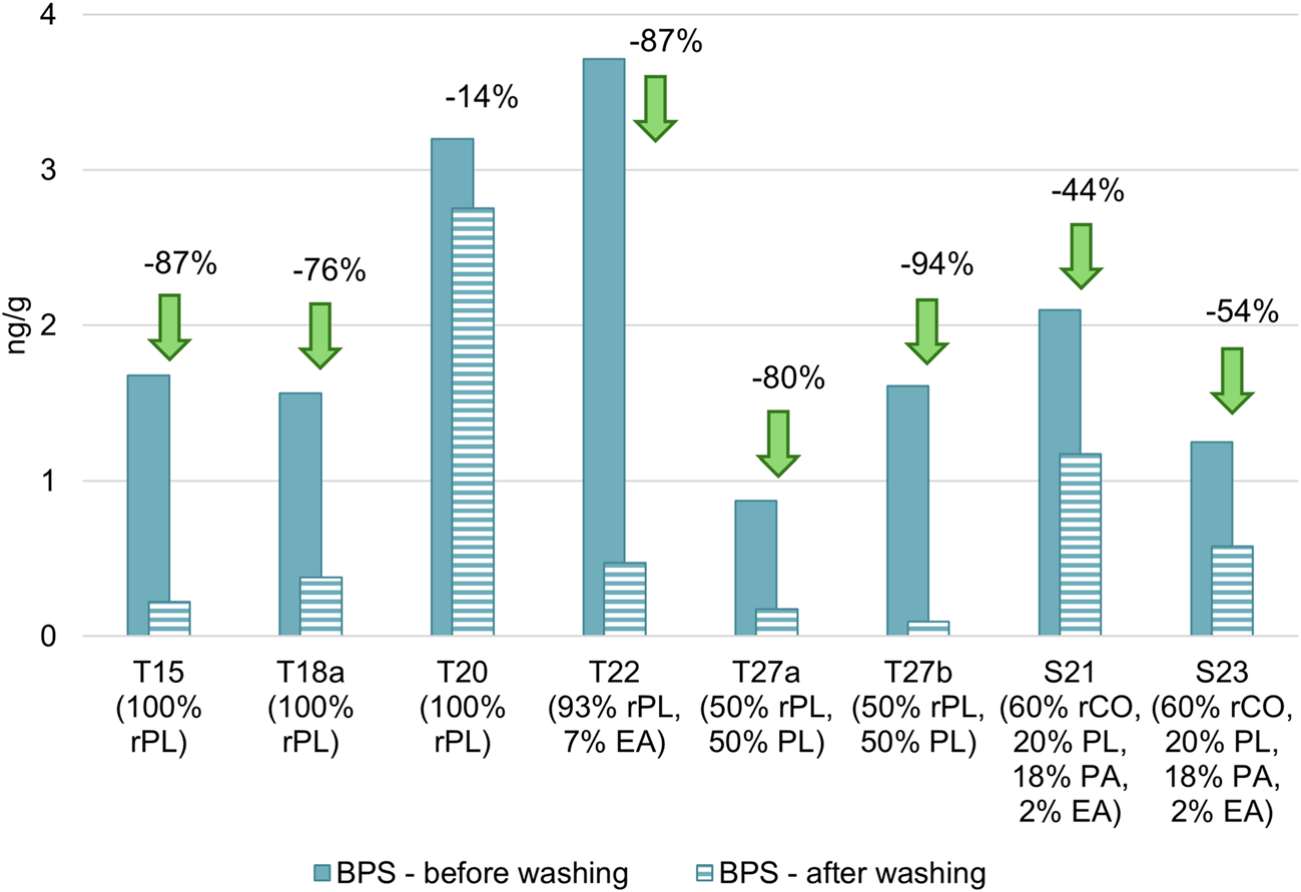
Decrease in BPS in new textiles after one wash cycle (a negative numerical value indicates a percentage decrease after a wash cycle, a positive value means an increase); rPL = recycled polyester, EA = elastane, PL = polyester, rCO = recycled cotton, PA = polyamide

As shown in Fig. 3 and Fig. 4, the data present a decrease in BPA (*p*-value = 0.0063) and BPS (*p*-value = 0.0028) levels after washing in seven out of eight samples, demonstrating that the washing process can effectively reduce the level of bisphenols in textiles. The reduction of BPS (approx. 40–90%) is more significant than that of BPA (approx. 30–70%). The different water solubility of these substances may explain this difference: 1,100 mg/L for BPS and 300 mg/L for BPA (Pivnenko et al. 2015). In contrast to these conclusions, sample T20 showed a slight increase (5%) in BPA concentration, while BPS concentration decreased by 14%. Considering the fairly consistent trends observed in the other textiles tested, we believe that the slight changes in concentrations in this sample can be attributed to measurement uncertainty (the expanded uncertainty of 20% was calculated with the coverage factor k = 2, providing an approximate 95% confidence level). This material, therefore, appears to be relatively inert regarding the effects of washing on the presence of bisphenols.

The behaviour of BPA in textiles during the washing process was also investigated by Wang et al. (2019). Our results support their findings regarding a decrease in BPA levels during washing. However, their results from the group washing several textiles together, which reflect real-life scenarios, suggest that cross-contamination during washing could potentially increase the BPA concentration in the textiles.

### Dermal exposure assessment

In our assessment of dermal exposure to bisphenols from the textile samples analysed, we initially assessed exposure from dry textiles. Using Eq. 1, we obtained the *EXPderm* values, which we present in Table 3 with the maximum, median and mean values for the T-shirts and socks made from conventional and recycled materials. Not surprisingly, these *EXPderm* values follow the same trend discussed above for the summarised results of the BPA and BPS analysis in textile samples (Table 1). Notably, the maximum exposure to BPA was significantly higher in recycled fabric T-shirts (209 pg/kg bw/day) than in other textiles. Conversely, BPS exposure was highest in socks made from conventional fabric (50.7 pg/kg bw/day).

**Table 3:** Summary dermal exposure doses (in pg/kg bw/day) calculated for a 70 kg adult in contact with the dry textiles

	Max EXPderm [pg/kg bw/day]	Median EXPderm [pg/kg bw/day]	Mean EXPderm [pg/kg bw/day]
All T-shirts (*n* = 33^a^)			
BPA	209	2.02	20.4
BPS	27.3	0.556	2.15
Conventional fabric T-shirts (*n* = 14)			
BPA	18.7	2.12	4.64
BPS	27.3	0.486	3.33
Recycled fabric T-shirts (*n* = 19^b^)			
BPA	209	1.26	31.9
BPS	6.01	0.580	1.28
All socks (*n* = 24^c^)			
BPA	12.8	0.248	1.23
BPS	50.7	0.086	3.00
Conventional fabric socks (*n* = 10)			
BPA	0.433	0.182	0.219
BPS	50.7	0.214	7.02
Recycled fabric socks (*n* = 14^d^)			
BPA	12.8	0.340	1.95
BPS	0.864	0.064	0.130

^a^33 samples made of 31 T-shirt items (4 samples were from 2 T-shirts)

^b^19 samples made of 17 T-shirt items (as above)

^c^24 samples made of 23 socks (2 samples were from 1 sock)

^d^14 samples made of 13 socks (as above)

Comparing our data with the current EFSA TDI value for BPA (0.2 ng/kg bw/day) (EFSA Panel on Food Contact Materials, Enzymes and Processing Aids 2023), we conclude that exposure to BPA from dry textiles generally does not exceed this limit. The only exception is the case of the maximum *EXPderm* for recycled T-shirts (209 pg/kg bw/day; sample T25; 62% recycled polyester, 33% viscose, 4% elastane), where the exposure value obtained corresponds to the value recommended by the EFSA.

Secondly, an experiment with sweat-soaked textiles was conducted for four samples to simulate a scenario where sweat could facilitate exposure to these substances. When selecting the samples for the experiment, we tried to consider different aspects – type of sample (socks vs. T-shirts), type of fabric (conventional vs. recycled) and various concentrations of the analytes in the samples—high (T29, BPA = 535 ng/g), but also close to the mean and median of BPS and BPA in the different sample categories to obtain the most comprehensive exposure estimation even with a small sample size. In the calculation (Eq. 2), the parameter *F*_*pen*_ was used, for which two recommended values are in the literature. Therefore, the values *EXPderm*_*wet-low*_ and *EXPderm*_*wet-high*_ are listed in Table 4. These data indicate that all textile samples in the wet state exceed the new TDI value for BPA. In the case of sample T29 (recycled T-shirt; 70% cotton, with 30% of it being recycled cotton, 28% recycled polyester, 2% elastane), the exposure exceeds the TDI by almost 125–570 times, depending on whether we consider the *EXPderm*_*wet-low*_ or *EXPderm*_*wet-high*_ value. Notably, the detected concentrations in these samples were not extreme (except for sample T29). In the case of sample S18 (recycled T-shirt), for example, the detected BPA concentration corresponds to the median value of the BPA concentration in recycled socks (16.6 ng/g).

**Table 4:** Summary of dermal exposure doses (in ng/kg bw/day) calculated for a 70 kg adult, considering contact with sweat-moistened textiles

	BPA			BPS		
Sample ID	Concentration in textile [ng/g]	EXPderm_wet-low_ [ng/kg bw/day]	EXPderm_wet-high_ [ng/kg bw/day]	Concentration in textile [ng/g]	EXPderm_wet-low_ [ng/kg bw/day]	EXPderm_wet-high_ [ng/kg bw/day]
T11^a^	54.0	1.80	8.29	36.7	9.93	45.7
T20^b^	32.1	1.42	6.51	3.20	1.65	7.58
T29^c^	535	24.8	114	14.3	1.45	6.67
S18^d^	16.1	0.223	1.03	12.1	0.053	0.246

^a^T11: 96% viscose, 4% elastane

^b^T20: 100% recycled polyester

^c^T29: 70% cotton (30% recycled cotton), 28% recycled polyester, 2% elastane

^d^S18: 54% bamboo viscose, 22% recycled polyester, 22% bio cotton, 2% elastane

It is worth noting that these data represent an estimate of exposure under extreme conditions (wet textiles), which could be significantly higher than under everyday conditions. However, the dermal route is traditionally considered a lower source of BPA exposure in units of percentage compared to dietary intake (Lu et al. 2018). Therefore, it is concerning that dermal exposure alone can reach or even exceed the EFSA TDI. Even though BPA exposure from textiles appears to be low compared to dietary sources, differences in metabolic processing and the longer retention in the body make dermal exposure more concerning than previously thought. The fact that dermally absorbed BPA remains in the body longer, with more remaining unconjugated (Liu and Martin 2017), suggests a potentially more significant biological impact than the same amount of ingested BPA.

## Conclusion

To the best of our knowledge, our study not only extends the limited dataset on the presence of bisphenols in everyday adult clothing but also provides the first data on the presence of bisphenols in clothing made from recycled materials. Due to increasing sustainability efforts, this sector has gained considerable attention and popularity in recent years. We have successfully developed and optimised a method for determining BPA, BPB, BPF and BPS in textiles and applied the method to 57 samples (24 conventional and 33 recycled) of clothing. Of all analytes monitored, BPA and BPS dominated with a large variability in detected concentrations (BPA range: < 0.050–625 ng/g; BPS range: 0.277–2,474 ng/g), with BPA levels increasing with decreasing cotton content. BPF was only detected in one sample, and BPB was not. The differences between the median concentrations, such as the BPA content in conventional textiles (7.66 ng/g) compared to recycled textiles (13.5 ng/g) and the BPS content in conventional textiles (3.42 ng/g) compared to recycled textiles (1.85 ng/g), indicate a notable shift in favour of BPS in conventional textiles, probably due to legislative pressure. These findings can be a sign of the adaptability of manufacturing practices and highlight the urgent need for further research on the impact of the recycling process on the levels of emerging chemicals in textiles. Our comprehensive dermal exposure assessment, considering both dry and sweat-wet textiles, shows that dermal exposure may exceed the new EFSA limit for BPA, especially under conditions like sweat-wet textiles. Although there are no restrictions or recommended limits for BPS exposure, the growing evidence of its biological effects similar to BPA's, associated with the increasing presence in the environment and consumer products, suggests that regulatory authorities should consider regulating this substance. In addition, our washing experiments demonstrate a decrease in bisphenols' presence after washing – an important observation for consumer safety. Consequently, we recommend that consumers wash new textile products at least once prior to use to reduce potential dermal exposure to bisphenols.

We acknowledge that our study has certain limitations, including the relatively limited number of textile samples and the inherent uncertainties in used dermal exposure assessment models, especially the one involving wet textiles, which represent extreme scenario. Future research directions could focus on increasing the sample size and diversity by including a broader range of clothing items beyond T-shirts and socks. Additionally, future studies could explore a broader spectrum of chemical contaminants present in textiles, recognising that individuals may be dermally exposed to a complex mixture of chemicals (flame retardants (brominated flame retardants or organophosphates), chlorinated paraffins or per- and polyfluoroalkylated substances). Our study specifically focused on bisphenols due to the limited research available on this topic, serving as a starting point for more comprehensive investigations. Future research could also extend laundry experiments to simulate real-life conditions better and investigate potential cross-contamination.

Our study, particularly concerning the presence of bisphenols in recycled textiles, contributes to understanding the distribution of bisphenols in textiles and emphasises the need for continuous monitoring and research into the health risks posed by bisphenols and other chemicals in textiles.

## Supplementary Information

Below is the link to the electronic supplementary material.

Supplementary file1 (DOCX 152 KB)

## Data Availability

The raw data will be available by the corresponding author upon reasonable request.
