# Supplementary material for: Bisphenols in daily clothes from conventional and recycled material: evaluation of dermal exposure to potentially toxic substances
Source: Environ Sci Pollut Res Int. 2024 Sep 6;31(43):55663–75. doi: 10.1007/s11356-024-34904-4 (PMC11415442; doi:10.1007/s11356-024-34904-4)
Supplement: Supplementary file 1 — Supplementary file1 (DOCX 152 KB) [file 11356_2024_34904_MOESM1_ESM.docx]

**SUPPLEMENTARY INFORMATION**

**Bisphenols in Daily Clothes from Conventional and Recycled Material: Evaluation of Dermal Exposure to Potentially Toxic Substances**

Jurikova M^a^., Dvorakova D.^a^, Bechynska K.^a^, Pulkrabova J.^a^*

^a^University of Chemistry and Technology (UCT), Prague, Faculty of Food and Biochemical Technology, 6 Department of Food Analysis and Nutrition, Technicka 5, 166 28, Prague, Czech Republic

* corresponding author (jana.pulkrabova@vscht.cz)

**Text S1** Catalogue numbers of utilized analytical standards

- Native analytical standards: BPA (catalogue no.: 42088), BPB (catalogue no.: 50877), BPF (catalogue no.: 51453); BPS (catalogue no.: 43034)
- Isotopically labelled analytical standards: d6-BPA (catalogue no.: 2417.15-K-IP), d8-BPS (catalogue no.: 12547.12-100-AN)

**Table S1** The content of the chemicals (in grams) used present in 1 liter of synthetic sweat^a^

| **Chemical** | **m [g]** |
| --- | --- |
| L-histidine monohydrochloride monohydrate (C_6_H_9_O_2_N_3_·HCl·H_2_O) | 0.5 |
| sodium chloride (NaCl) | 5 |
| sodium dihydrogen phosphate dihydrate (NaH_2_PO_4_·2H_2_O) | 2.2 |

^a^Solution was subsequently adjusted to a 5.5 (± 0.2) pH with NaOH solution (0.1 mol/L)

**Table S2** List of analyzed textile samples and additional information (CO – cotton; CLY – lyocell; EA – elastane; PA – polyamide; PL – polyester; VI – viscose; rPL – recycled polyester; rCO - recycled cotton; oCO – organic cotton; bioCO – bio cotton; rPA – recycled polyamide)

| **Sample** |  | **Textile composition** | **Type** | **Color** | **Country of origin** |
| --- | --- | --- | --- | --- | --- |
| S1 | CONVENTIONAL TEXTILE | 74% CO 23% PL 2% EA 1% PA | socks | white | Turkey |
| S2 |  | 76% CO 22% PA 2% EA | socks | purple unicorns print | Vietnam |
| S3 |  |  | socks | pink | Vietnam |
| S4 |  |  | socks | white | Vietnam |
| S5 |  | 77% PL (77) 21% PA (21) EA (EA) | socks | yellow | Pakistan |
| S6 |  |  | socks | grey | Pakistan |
| S7 |  | 98% PL 2% EA | socks | green | x |
| S8 |  |  | socks | yellow | x |
| S9 |  |  | socks | red | x |
| S10 |  | NA | socks | red cherries | x |
| T1 |  | 100% CO | T-shirt | white | Turkey |
| T2 |  |  | T-shirt | red | x |
| T3 |  |  | T-shirt | blue-white | x |
| T4 |  |  | T-shirt | blue | x |
| T5 |  |  | T-shirt | orange | India |
| T6 |  |  | T-shirt | Spiderman print | India |
| T7 |  |  | T-shirt | black | x |
| T8 |  |  | T-shirt | Heisenberg print | Bangladesh |
| T9 |  | 100% PL | T-shirt | blue | China |
| T10 |  | 74% PL 19% CLY 7% EA | T-shirt | grey-pink | Vietnam |
| T11 |  | 96% VI  4% EA | T-shirt | floral print | Turkey |
| T12 |  | NA | T-shirt | sunset print | x |
| T13 |  | NA | T-shirt | Homer print | x |
| T14 |  | NA | T-shirt | blue dolphins print | x |
| T15 | RECYCLED TEXTILE | 100 % rPL | T-shirt | colorful | India |
| T16 |  |  | T-shirt | white | x |
| T17 |  |  | T-shirt | light blue | Thailand |
| T18a |  |  | T-shirt | colorful, transparent | India |
| T18b |  |  |  | green |  |
| T19 |  |  | T-shirt | white | Zimbabwe |
| T20 |  |  | T-shirt | blue | Czech Republic |
| T21 |  |  | T-shirt | white | Zimbabwe |
| T22 |  | 93% rPL 7% EA | T-shirt | orange | Cambodia |
| T23 |  |  | T-shirt | blue | Cambodia |
| T24 |  | 60 % rPL 40% CO | T-shirt | light green | x |
| T25 |  | 62% rPL 33% VI 5% EA | T-shirt | burgundy | Bangladesh |
| T26 |  | 55% rPL 45% PL | T-shirt | orange | China |
| T27a |  | 50% rPL 50% PL | T-shirt | green with flower print, transparent | China |
| T27b |  |  |  | green |  |
| T28 |  | 58% rCO 38% rPL 4% EA | T-shirt | blue | China |
| T29 |  | 70% CO (30% from that is rCO) 28% rPL 2% EA | T-shirt | light brown | x |
| T30 |  | 91% rPL 9% EA | T-shirt | pink | x |
| T31 |  | 95 % CO (71% of that is oCO, 24% rCO) 5% EA | T-shirt | brown | x |
| S11 |  | 98% rPL 2% EA | Socks | striped | x |
| S12a |  | 49% rPL 48% CO 2% EA 1% rPA | Socks | white | Turkey |
| S12b |  |  |  | grey |  |
| S13 |  | 58% CO 39% rPL 2% EA 1% rPA | Socks | white | x |
| S14 |  | 64% CO 32% rPL 3% EA 1% PA | Socks | black | x |
| S15 |  |  | Socks | pink | x |
| S16 |  |  | Socks | white | x |
| S17 |  | 54% bamboo VI 22% rPL 22% bioCO 2% EA | Socks | hearts print | Czech Republic |
| S18 |  |  | Socks | pictures print | United Kingdom |
| S19 |  | 80% rCO 17% PL 3% EA | Socks | burgundy | Turkey |
| S20 |  | 60% rCO 20% PL 18% PA 2% EA | Socks | white | Turkey |
| S21 |  |  | Socks | red | Turkey |
| S22 |  |  | Socks | pink | Turkey |
| S23 |  |  | Socks | beige | Turkey |

NA = information not available

**Table S3** Performance characteristics of SPE extraction of bisphenols (sweat leaching experiment); validated at concentration 16 ng/mL of sweat

| **Analyte** | **Recovery (%)** | **RSD (%)** | **LOQ (ng/mL of sweat)** |
| --- | --- | --- | --- |
| BPA | 94 | 4 | 0.02 |
| BPS | 112 | 2 | 0.02 |
| BPF | 105 | 3 | 0.2 |
| BPB | 93 | 9 | 0.2 |

**Table S4** UHPLC-MS/MS mobile phase gradient

| **Time [min]** | **A [%]** | **B [%]** | **Flow [mL/min]** |
| --- | --- | --- | --- |
| 0.0 | 90 | 10 | 0.3 |
| 2.0 | 40 | 60 | 0.3 |
| 6.0 | 0 | 100 | 0.4 |
| 6.1 | 90 | 10 | 0.4 |
| 8.0 | 90 | 10 | 0.4 |

A = 0.05 % CH_3_COOH in deionized water; B = 0.05 % CH_3_COOH in MeOH

**Table S5** Target bisphenols and optimized LC-MS/MS parameters

| **Analyte** | **Abbreviation** | **CAS number** | **Retention time**  **[min]** | **Parent ion (*m/z*)** | **Product ion 1** | | **Product ion 2** | | **Product ion 3** | |
| --- | --- | --- | --- | --- | --- | --- | --- | --- | --- | --- |
|  |  |  |  |  | **(*m/z*)** | **Collision (V)** | **(*m/z*)** | **Collision (V)** | **(*m/z*)** | **Collision (V)** |
| 4,4′-Sulfonyldiphenol | BPS | 80-09-1 | 3.1 | 249 | 108 | 32 | 156 | 20 | 92.1 | 40 |
| Bis(4-hydroxyphenyl)methane | BPF | 620-92-8 | 3.6 | 199 | 93 | 20 | 105 | 24 | 77 | 24 |
| 2,2-Bis(4-hydroxyphenyl)propane | BPA | 80-05-7 | 4.0 | 227.1 | 212 | 20 | 133 | 24 | x | x |
| 2,2-Bis(4-hydroxyphenyl)butane | BPB | 77-40-7 | 4.3 | 241.1 | 226 | 20 | 211 | 28 | 117 | 52 |
| Bisphenol S-d8 (2,2',3,3',5,5',6,6'-d8) | d6-BPA | 86588-58-1 | 3.1 | 257 | 112 | 32 | 160 | 24 | 96.1 | 40 |
| Bisphenol A-d6 (dimethyl-d6) | d8-BPS | 92739-58-7 | 4.0 | 233 | 215 | 20 | 138 | 32 | x | x |

x = not monitored


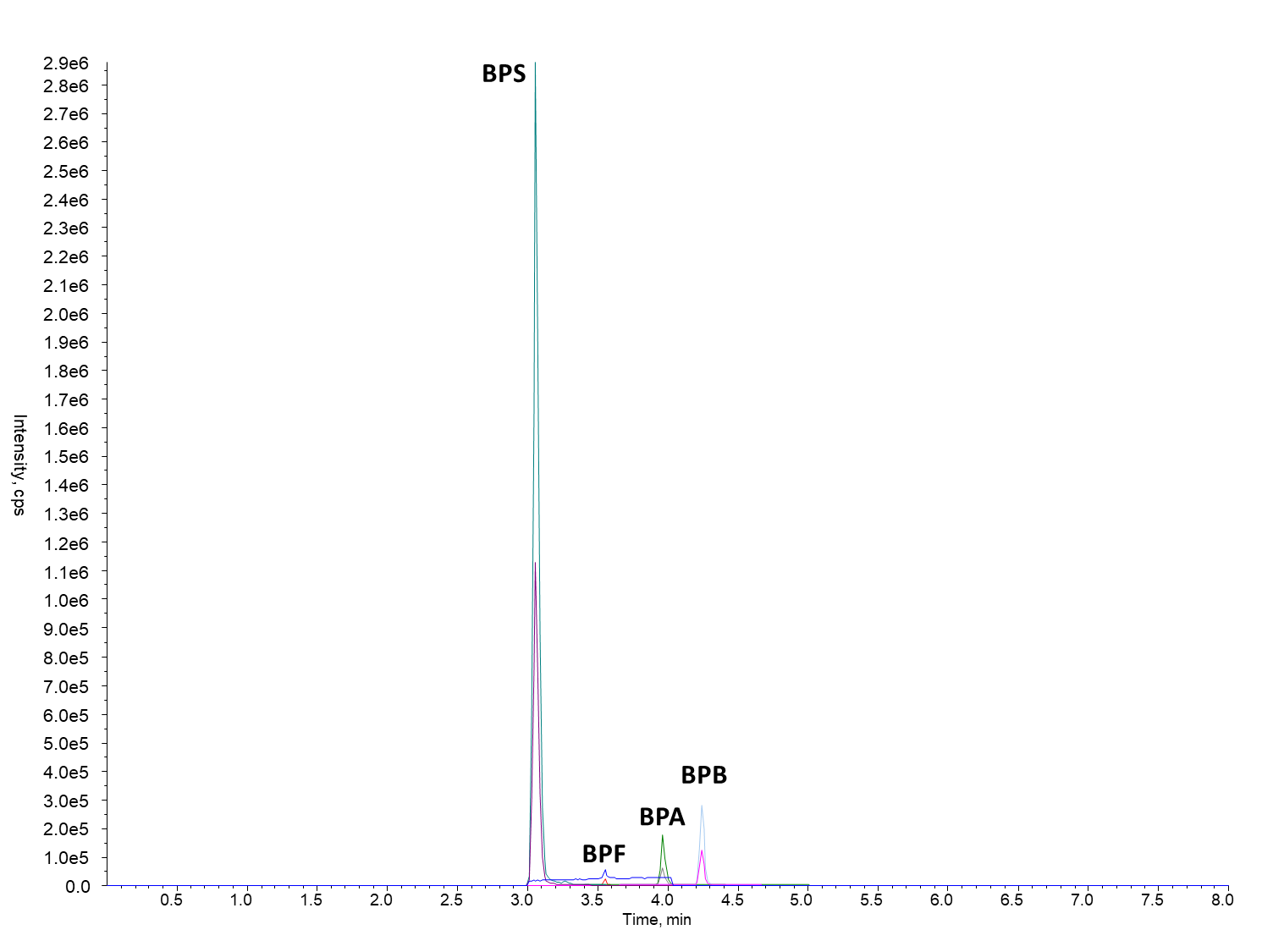


**Figure S1** Extracted ion UHPLC-MS/MS chromatogram of target bisphenols in solvent standard (10 ng/ml)


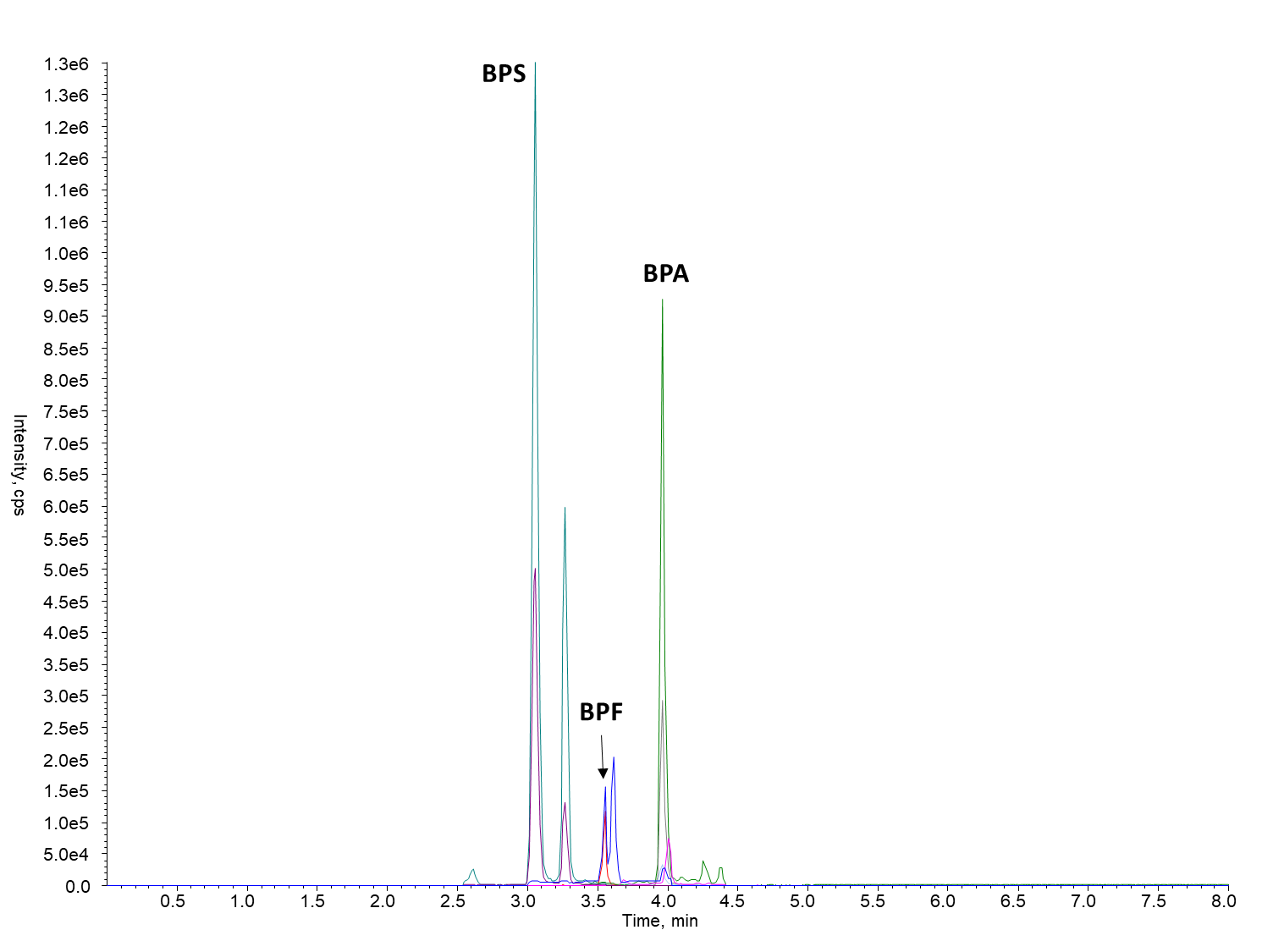


**Figure S2** Extracted ion UHPLC-MS/MS chromatogram of target bisphenols in sample T20 (recycled T-shirt; 100% recycled polyester)

**Figure S3** Comparison of the extraction efficiency of tested solvents by extracting recycled fabric bag as test material (in three repetition for each solvent) – median concentrations (in ng/g) detected after 2 hours UAE. The error bars represent standard deviations between the individual samples.

**Figure S4** Relative recovery (%) of repeated extraction of a recycled fabric bag sample (one sample in three parallel experiments A1, A2, A3)
